# TaBZR1 Directly Activates *Autophagy‐Related (ATG) 8* to Promote Wheat Defence to Powdery Mildew

**DOI:** 10.1111/pbi.70403

**Published:** 2025-11-17

**Authors:** Yamei Zhuang, Yongbo Li, Yulian Li, Wang Chen, Ran Han, Xiaolu Wang, Kai Wang, Wenjing Xu, Qingqi Fan, Jianjun Liu, Takao Komatsuda, Huan Chen, Cheng Liu, Guang Qi

**Affiliations:** ^1^ Crop Research Institute, Shandong Academy of Agricultural Sciences/National key Laboratory of Wheat Improvement Jinan China; ^2^ School of Agriculture and Biology Shanghai Jiao Tong University Shanghai China

**Keywords:** autophagy, brassinosteroid, powdery mildew, TaATG8g, TaBZR1, *Triticum aestivum*

1

Common wheat ( *Triticum aestivum* , AABBDD) is an important staple crop for approximately 35% of the global population. The powdery mildew disease caused by *Blumeria graminis* f. sp. *tritici* (*Bgt*) leads to significant yield losses in most wheat‐producing regions (Zorb et al. 2018). To effectively cope with this disease, sustained efforts are being committed to mine more effective *Pm* genes and understand the mechanisms of the *Bgt*–wheat interaction. Autophagy is a conserved cellular recycling process to maintain cellular homeostasis. We previously revealed that Autophagy‐related (ATG) 8, a key regulator in autophagy, is involved in grain development in wheat (Li et al. 2021). In this study, we studied the function of *TaATG8g* in regulating wheat defence against *Bgt*, and identified TaBZR1, a key transcription factor in BR signalling (Lyu et al. 2024), as a positive regulator of *ATG8g* expression. Finally, we established a TaBZR1‐*TaATG8g* gene module that plays critical roles in wheat resistance against *Bgt*.

To investigate the roles of TaATG8g in disease resistance, we examined the expression of *TaATG8g* in response to *Bgt* infection in both compatible and incompatible interactions using F_2:6_ recombinant inbred lines (RILs) derived from the cross of Yannong 1212 (susceptible to *Bgt*) and Citr 17 345 (carrying *Pm21*). Using qRT‐PCR, we found that *TaATG8*g expression significantly increased in the resistant RILs (RIL‐R) but not in the susceptible RILs (RIL‐S) when inoculated with the *Bgt* isolate E09 (Figure 1a and Figure S1a). Consistently, immunoblotting analysis revealed the increase of TaATG8g and TaATG8‐phosphatidylethanolamine (PE) conjugate (TaATG8‐PE) in the incompatible interaction (Figure 1b and Figure S2). In agreement with the above findings, lowering *TaATG8g* expression by BSMV‐mediated VIGS promoted susceptibility to *Bgt* in Fielder (Figure 1c–e). We next generated a knockout mutant of *TaATG8g* using CRISPR/Cas9‐mediated genome editing. Consistently, *TaATG8g* mutants (*TaATG8g‐KO*) showed significantly reduced resistance against *Bgt* (Figure 1f,g and Figure S2). Further we generated transgenic lines overexpressing *TaATG8g* driven by the maize *Ubiquitin* promoter in Fielder. Two stable T3 transgenic lines (*TaATG8g*‐*OE1* and *TaATG8g*‐*OE3*) (Figure S4a) showed significantly reduced *Bgt* growth and development relative to WT (Figure 1f,g and Figure S1b,c). Additionally, the OE lines displayed similar growth and grain yield traits as WT, suggesting that overexpression of *TaATG8g* has no adverse effects on wheat agronomic characteristics (Figure S5).

**FIGURE 1:**
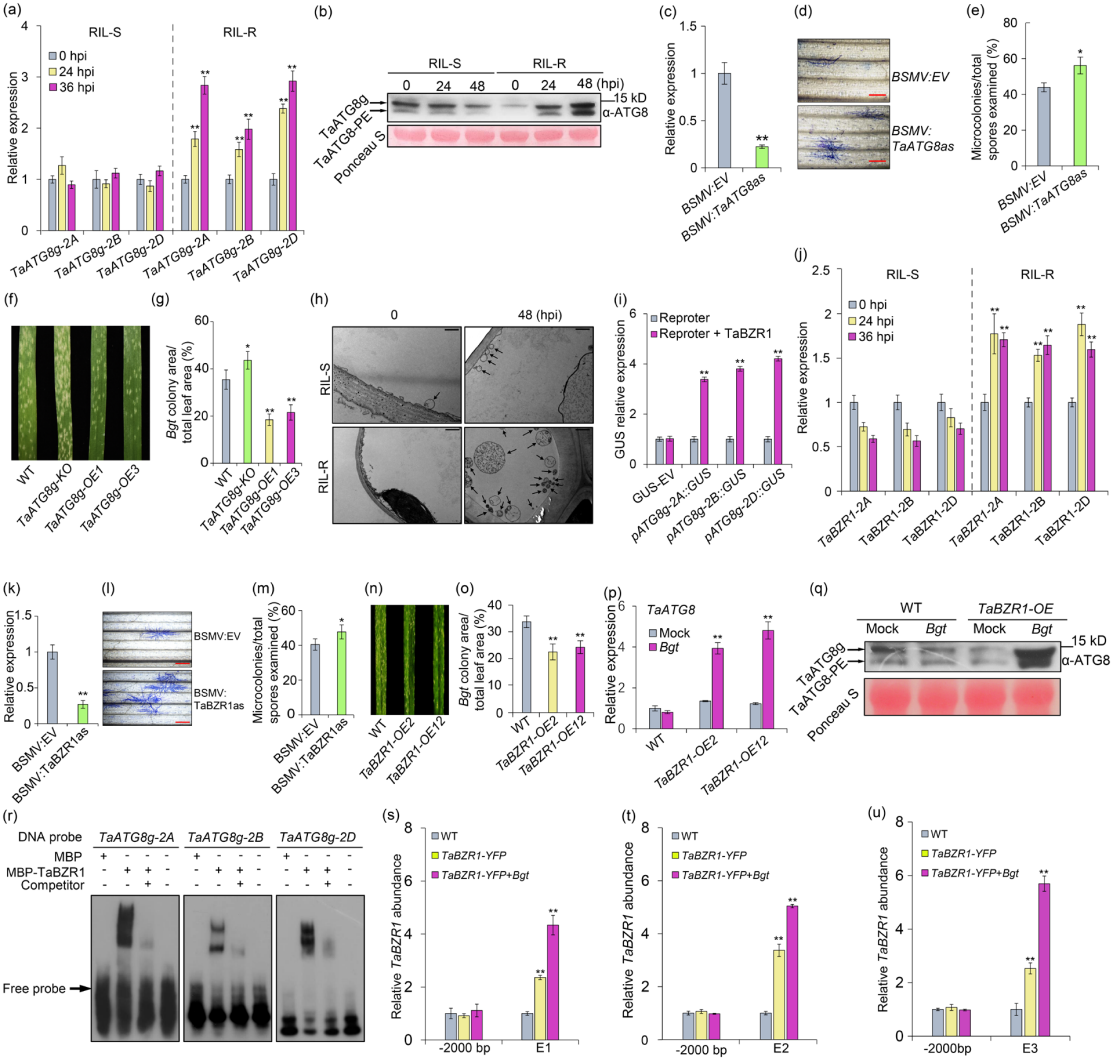
Functional characterisation of TaBZR1‐TaATG8g module‐mediated resistance against powdery mildew. (a, b) *Bgt‐*induced TaATG8g in compatible and incompatible interactions. Increased *TaATG8g* transcript levels, TaATG8g and TaATG8‐PE in the RIL‐R but not in the RIL‐S were revealed by qRT‐PCR (a) and immunoblotting (b). (c–e) Enhanced development of *Bgt* on leaves of *TaATG8g* silenced plants. Recombinant virus BSMV:TaATG8as was used for silencing *TaATG8g*, with BSMV:EVC, an empty BSMV vector, served as a control (c). Lowering transcription of *TaATG8g* increased the development of *Bgt* as shown by Coomassie blue staining (d) and quantitative comparison with WT (e). (f, g) *Bgt* growth indicated by the photographs of *Bgt*‐infected leaves (f) and quantitative comparison of the percentages of *Bgt* colony area (g). (h) TEM observation of autophagic structures (arrows) in the *Bgt*‐infected susceptible and resistant RILs. Scale bar, 10 μm. (i) Transient expression assays of *TaATG8g* promoter‐driven GUS and TaBZR1 in *Nicotiana benthamiana* protoplasts. LUC expression was used as an internal control. (j) *Bgt*‐induced expression of *TaBZR1* in compatible and incompatible interactions. (k–m) Development of *Bgt* on *TaBZR1* silenced leaves. Silencing *TaBZR1* was achieved as described in (c). Increased *Bgt* microcolonies development in *TaBZR1* silenced leaves was revealed by Coomassie blue staining (l) and quantitative comparison with WT (m). (n, o) *Bgt* colony growth among WT and *TaBZR1* OE lines. Phenotypes were recorded as described above. (p–r) *Bgt*‐induced *TaATG8g* expression in *TaBZR1* OE lines. qRT‐PCR (p) and immunoblotting (q) indicated the increased expression of *TaATG8g* in *TaBZR1* overexpressing lines. (s–v) Direct binding of *TaATG8g* promoter regions by TaBZR1 in vitro and in vivo, as indicated by EMSA (s) and ChIP–qPCR assays (t–v). The datasets shown are all typical of three independent experiments. Error bars indicate standard deviation; *p < 0.05, **p < 0.01, according to Student's *t*‐test.

ATG8 family proteins play essential roles in autophagosome biogenesis; we therefore tested the potential role of autophagy in wheat resistance against *Bgt*. Transmission electron microscopy (TEM) observation showed that autophagic bodies significantly increased in the RIL‐R seedlings but not in the RIL‐S seedlings (Figure 1h). In addition, Exogenous application of LiCl (an autophagy activator) significantly reduced susceptibility to *Bgt*, while the resistance of wheat to *Bgt* was reduced when 3‐Methyladenine (3‐MA, an inhibitor of autophagy) was applied (Figure S2a–d) (Yang et al. 2013). As revealed by immunoblotting, TaATG8g and TaATG8‐PE significantly increased under LiCl treatment while depressed under 3‐MA treatment (Figure S2e). Together, these results indicated that *TaATG8g* positively regulates wheat resistance to *Bgt*.

To identify the transcription factors (TFs) regulating *TaATG8g* expression, we performed a yeast‐one‐hybrid screen using the *TaATG8g* promoter as bait. *TaBZR1* (*TraesCS2A02G187800*), a brassinazole‐resistant (BZR) TF, was found to be capable of binding to the promoter of *TaATG8g*. Further sequence analysis revealed that the promoter of *TaATG8g‐2A*, *‐2B*, and *‐2D* contain E‐boxes (CANNTG) and BRRE (CGTGT/CG) elements which were enriched in BZR1/BES1 targets (Yu et al. 2011) (Figure S3a). Moreover, in the transient expression assays with the promoter region of *TaATG8g‐2A*, *TaATG8g‐2B*, or *TaATG8g‐2D* fused to the β‐glucuronidase gene as reporters and *p35S::TaBZR1* as an effector (Figure S3b), TaBZR1 expression significantly increased the promoter activity of *TaATG8g* (Figure 1i). These results suggested that the increased transcription of *TaATG8g* in *Bgt*‐infected plants may be regulated by TaBZR1. We thus focused on analyzing the function of TaBZR1 in wheat defense against *Bgt*.

Similar to *TaATG8g*, *TaBZR1* expression significantly increased in the incompatible interaction (Figure 1j and Figure S1b). Lowering *TaBZR1* expression using BSMV‐mediated VIGS led to significantly increased microcolony development (Figure 1k–m). Subsequently, two independent transgenic lines (*TaBZR1‐OE2* and ‐*OE12*) (Lyu et al. 2024). Showed significantly reduced *Bgt* growth and development (Figure 1n,o and Figure S4a,b). Moreover, *Bgt*‐induced expression of *TaATG8g* was attenuated in the *TaBZR1*‐silenced plants but increased in the *TaBZR1* overexpressing lines (Figure 1p,q and Figure S9). Consistently, increased autophagic structures were observed in the *TaBZR1* overexpressing line (Figure 1r). These results suggested that TaBZR1 enhanced wheat defence against *Bgt* by upregulating *TaATG8g* expression.

To further elucidate whether TaBZR1 might directly bind to the promoter of *TaATG8g*, we performed electrophoretic mobility shift assays (EMSA). As anticipated, the MBP‐TaBZR1 protein was able to bind all three promoter fragments (Figure 1s). To verify whether TaBZR1's binding to *TaATG8g* promoters in planta, we next carried out chromatin immunoprecipitation quantitative PCR (ChIP–qPCR) assays using the transgenic *pUbi:TaBZR1‐YFP* plants. The ChIP–qPCR result showed higher enrichment of TaBZR1 in the promoter of *TaATG8g*, which is further boosted by *Bgt*‐infection (Figure 1t–v and Figure S3b). These results indicate that TaBZR1 can directly target and activate the expression of *TaATG8g*. Therefore, *TaBZR1* and *TaATG8g* comprise a functional module to promote wheat resistance against *Bgt*.

Our previous study indicated that BZR1 positively regulates effector‐triggered immunity in *Arabidopsis* (Qi et al. 2021). These findings prompted us to test whether TaBZR1 and TaATG8g promote wheat defence against *Bgt* through induction of reactive oxygen species (ROS) and cell death. We therefore stained the *Bgt*‐infected leaf using diaminobenzidine (DAB) and trypan blue (TPN) to visualise hydrogen peroxide (H_2_O_2_) accumulation and cell death. Compared to WT, significantly increased accumulation of H_2_O_2_ and cell death was shown in the *Bgt*‐inoculated *TaBZR1* and *TaATG8g* overexpressing lines (Figure 1w). To gain insight into the biological processes and genes regulated by TaATG8‐mediated autophagy in wheat defence against *Bgt*, RNA‐Seq was performed with the *TaATG8g* OE lines. A battery of differentially expressed genes was revealed (Figure S11a,b), among which three homeologous AP2/ERF TF genes were validated by qRT‐PCR to be upregulated in the *TaATG8g* OE lines in response to *Bgt* infection. Additional qRT‐PCR assay confirmed that they were depressed in the *TaATG8g‐KO* mutant or by silencing *TaBZR1* in response to *Bgt* infection (Figure S11c,d), suggesting that they act downstream of the TaBZR1‐*TaATG8g* module. The Arabidopsis ortholog of these ERF genes, that is, *AtERF11*, was demonstrated to promote ROS accumulation in salicylic acid (SA)/ethylene (ET)‐regulated plant resistance (Zheng et al. 2019), implying that TaATG8‐mediated autophagy may promote ROS accumulation through SA/ET signalling in wheat resistance against *Bgt*. Additionally, *Bgt*‐induced expression of *TaPR1* was shown to be significantly increased in the *TaBZR1‐* and *TaATG8g‐overexpressing* lines (Figure S5). These results suggest that TaBZR1‐ and TaATG8g‐promoted wheat resistance to *Bgt* might be associated with H_2_O_2_ accumulation, induced cell death, and enhanced *PR1* expression.

Searching for broad‐spectrum and durable resistance genes is important for wheat breeding. Overexpression of *TaBZR1* and *TaATG8g* both leads to increased resistance against *Bgt*. However, when infected by a field‐collected *Bgt* population, the OE lines of *TaATG8g* and *TaBZR1* showed attenuated resistance compared to that against E09 (Figure S13). This result suggested that TaATG8g‐ and TaBZR1‐mediated resistance was race‐specific, making it unsuitable for direct use in breeding. We can therefore pyramid TaATG8g or TaBZR1 with other resistance alleles to develop crops with broad‐spectrum resistance. Overall, this study provides new information for the genetic basis of wheat resistance against *Bgt*, and provides candidate genes with potential for wheat breeding.

## Author Contributions

G.Q., C.L., and H.C. designed the research, analyzed data, and wrote the manuscript. Y.Z. and Y‐L.L. Y.L. performed all experiments. W.C. performed the RNA‐Seq analysis. R.H. assisted with the generation of *TaATG8g* overexpressing lines. X.W., K.W., W.X. conducted VIGS and gene expression analysis. Q.F., J.L., and T.K. assisted in *Bgt* phenotyping and provided critical discussion. All authors read and approved the final manuscripts.

## Supporting information


**Data S1:** Supporting Information.

## Data Availability

The data that supports the findings of this study is available in the Supporting Information—S1 of this article.
