# Supplementary material for: TaBZR1 Directly Activates Autophagy‐Related (ATG) 8 to Promote Wheat Defence to Powdery Mildew
Source: Plant Biotechnol J. 2025 Nov 17;24(4):2018–20. doi: 10.1111/pbi.70403 (PMC13140519; doi:10.1111/pbi.70403)
Supplement: Supplementary file 1 — Data S1: Supporting Information. [file PBI-24-2018-s001.docx]

**TaBZR1 directly activates *autophagy-related (ATG) 8* to promote wheat defense to powdery mildew**

Yamei Zhuang^1†^, Yongbo Li^1†^, Yulian Li^1†^, Wang Chen^2^, Ran Han^1^, Xiaolu Wang^1^, Kai Wang^1^, Wenjing Xu^1^, Qingqi Fan^1^, Jianjun Liu^1^, Takao Komatsuda^1^, Huan Chen^2*^, Cheng Liu^1*^, and Guang Qi^1*^

**Supporting information**

**SI-1 Supplementary figures**

**Figure S1.** *Bgt-*induced expression of *TaATG8g* and *TaBZR1* in compatible and incompatible interactions.

**Figure S2.** *Bgt*-induced accumulation of TaATG8 protein in compatible and incompatible interactions.

**Figure S3.** Generation of *TaATG8g* mutant in common wheat using CRISPR/Cas9-mediated genome editing.

**Figure S4.** Effects of knockout and overexpression of *TaATG8* on wheat defense against *Bgt*.

**Figure S5.** Growth and grain traits of *TaATG8g* overexpression lines.

**Figure S6.** Effect of exogenous application of LiCl or 3-Methyladenine (3-MA) on wheat resistance to *Bgt*.

**Figure S7.** Structure of the *TaATG8* promoter and combinations of reporters and effector used in the transient expression assays.

**Figure S8.** Effects of overexpressing *TaBZR1* on wheat defense against *Bgt*.

**Figure S9.** Transcript level of *TaATG8* in leaves of *TaBZR1* silenced plants.

**Figure S10.** TEM observation of autophagic structures in *TaBZR1-OE12*.

**Figure S11.** Detection of H_2_O_2_ accumulation and cell death in the *Bgt*-infected leaves of *TaBZR1*-KO and *TaBZR1*-OE lines.

**Figure S12.** Detection of H_2_O_2_ accumulation and cell death in the *Bgt*-infected leaves of WT and *TaBZR1*-OE lines.

**Figure S13.** Analysis of the genes acted downstream of TaBZR1-*TaATG8g* module responding to *Bgt* inoculation.

**Figure S14.** Transcript level of *TaPR1* in leaves of *TaATG8-* and *TaBZR1-* OE lines relative to WT.

**Figure S15.** Infection phenotype of *TaATG8g-OE* and *TaBZR1-OE* lines to divergent Bgt isolates.

**SI-2 Supplementary materials and methods**


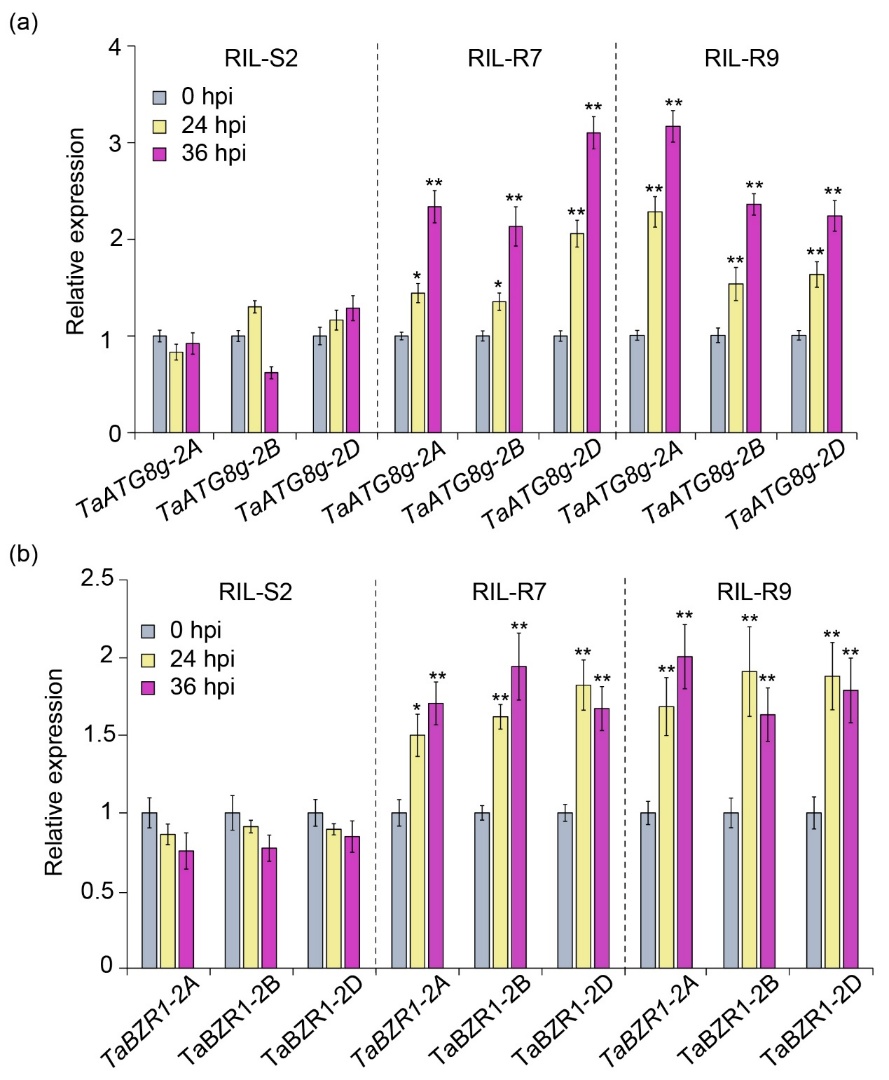


**Figure S1.** *Bgt-*induced expression of *TaATG8g* and *TaBZR1* in compatible and incompatible interactions.

Relative transcript levels of *TaATG8g* (a) and *TaBZR1* (b) increased in the resistant RILs (RIL-R7, 9) but not in the susceptible RIL (RIL-S2) revealed by qRT-PCR. Error bars indicate standard deviation calculated from at least three biological replicates. *, P < 0.05; **, P < 0.01 (Student’s *t*-test).


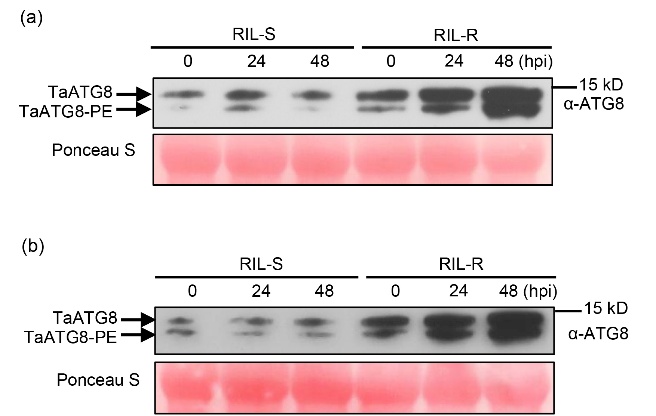


Figure S2. *Bgt*-induced accumulation of TaATG8 protein in compatible and incompatible interactions.

Protein levels of TaATG8 and TaATG8-PE increased in the resistant RILs (RIL-R) but not in the susceptible RILs (RIL-S) when infected by *Bgt*. Repeated immunoblotting assays (a, b) in different RIL-S and RIL-R lines were performed with the anti-TaATG8g antibody.


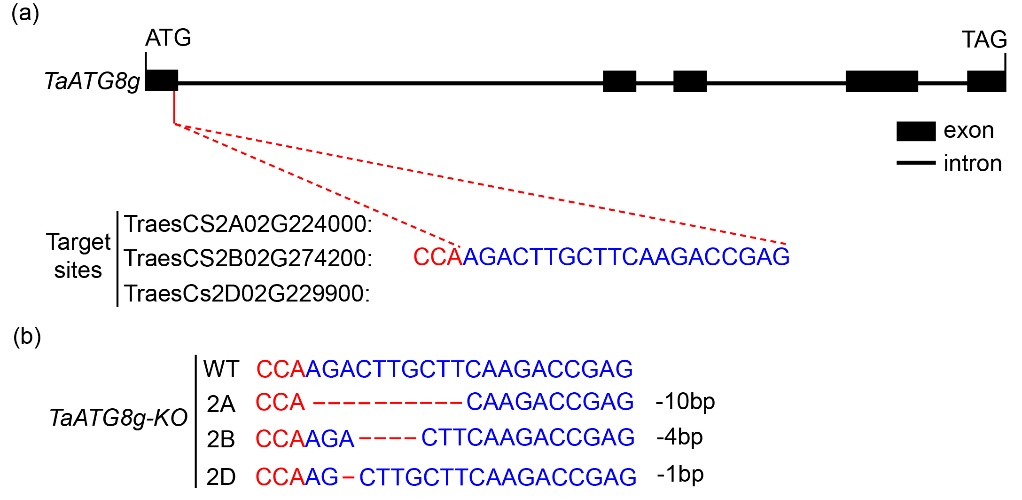


**Figure S3.** Generation of *TaATG8g* mutant in common wheat using CRISPR/Cas9-mediated genome editing.

(a) Schematic illustration of gene structure and sgRNA selection in *TaATG8g*. (b) The mutation sites of *TaATG8g*. Red, protospacer-adjacent motif (PAM); blue, sgRNA target sequence; “‐”, nucleotide deletions.


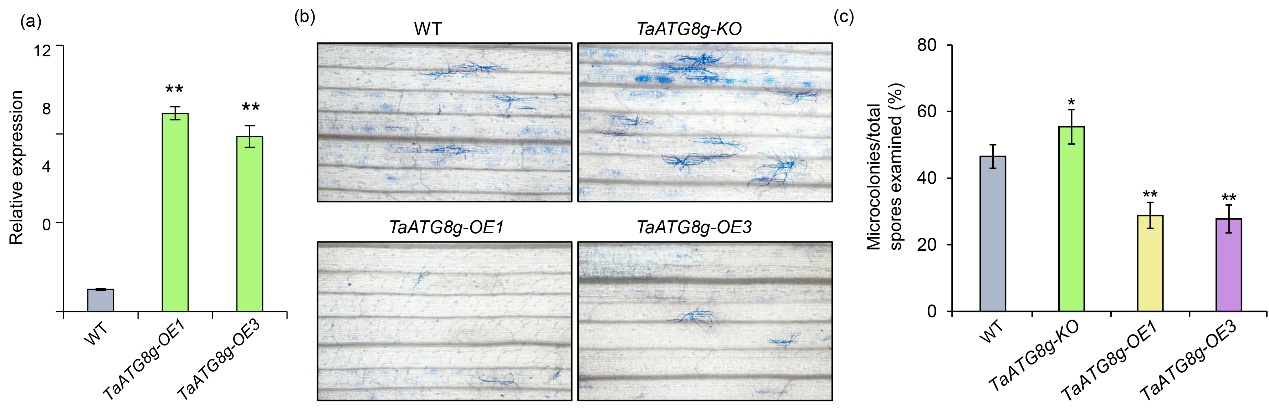


**Figure S4.** Effects of knockout and overexpression of *TaATG8* on wheat defense against *Bgt*.

(a) Relative expression of *TaATG8g* in two OE lines relative to WT revealed by qRT-PCR. (b, c) Knockout and overexpression of *TaATG8* depressed and increased *Bgt* microcolonies development, respectively, as shown by Coomassie blue staining (b) and quantitative comparison with the WT control (c). Error bars indicate standard deviation calculated from at least three biological replicates. *, P < 0.05; **, P < 0.01 (Student’s *t*-test).


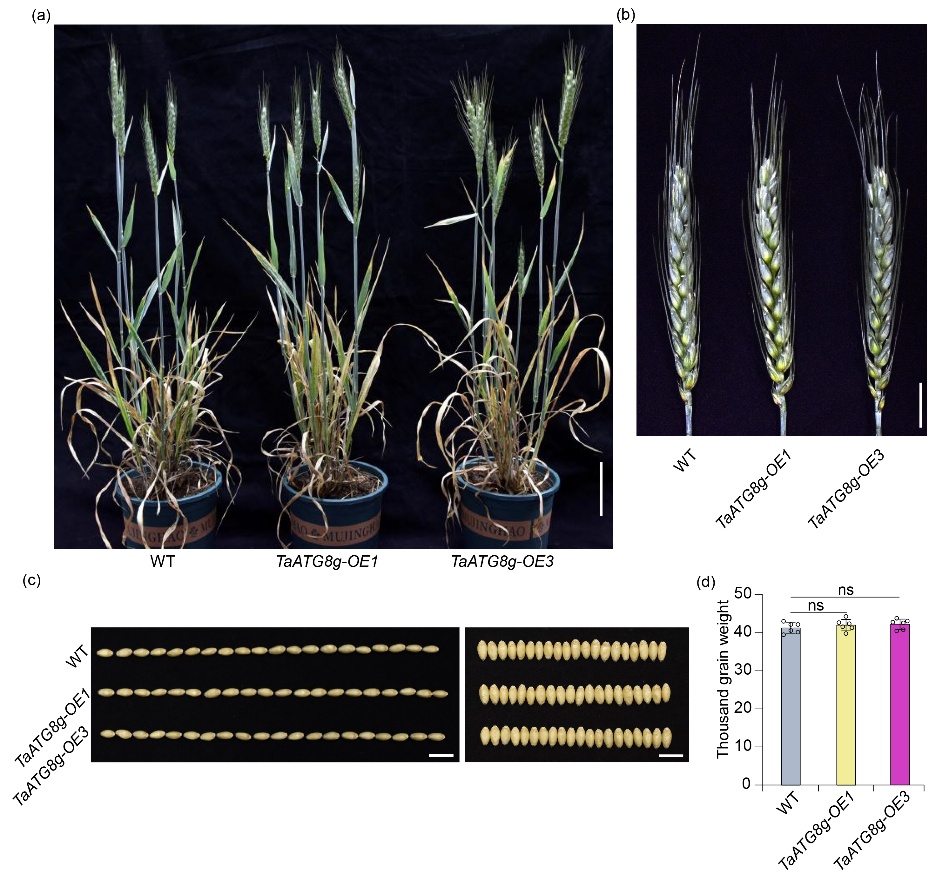


**Figure S5.** Growth and grain traits of *TaATG8g* overexpression lines.

Wheat plant (a) and spike (b) phenotype of *TaATG8g* OE lines grown in the greenhouse. (c, d) grain morphology and 1,000‐grain weights of *TaATG8g* OE lines. Scale bars: 10 cm (a), 2 cm (b), 1 cm (c).


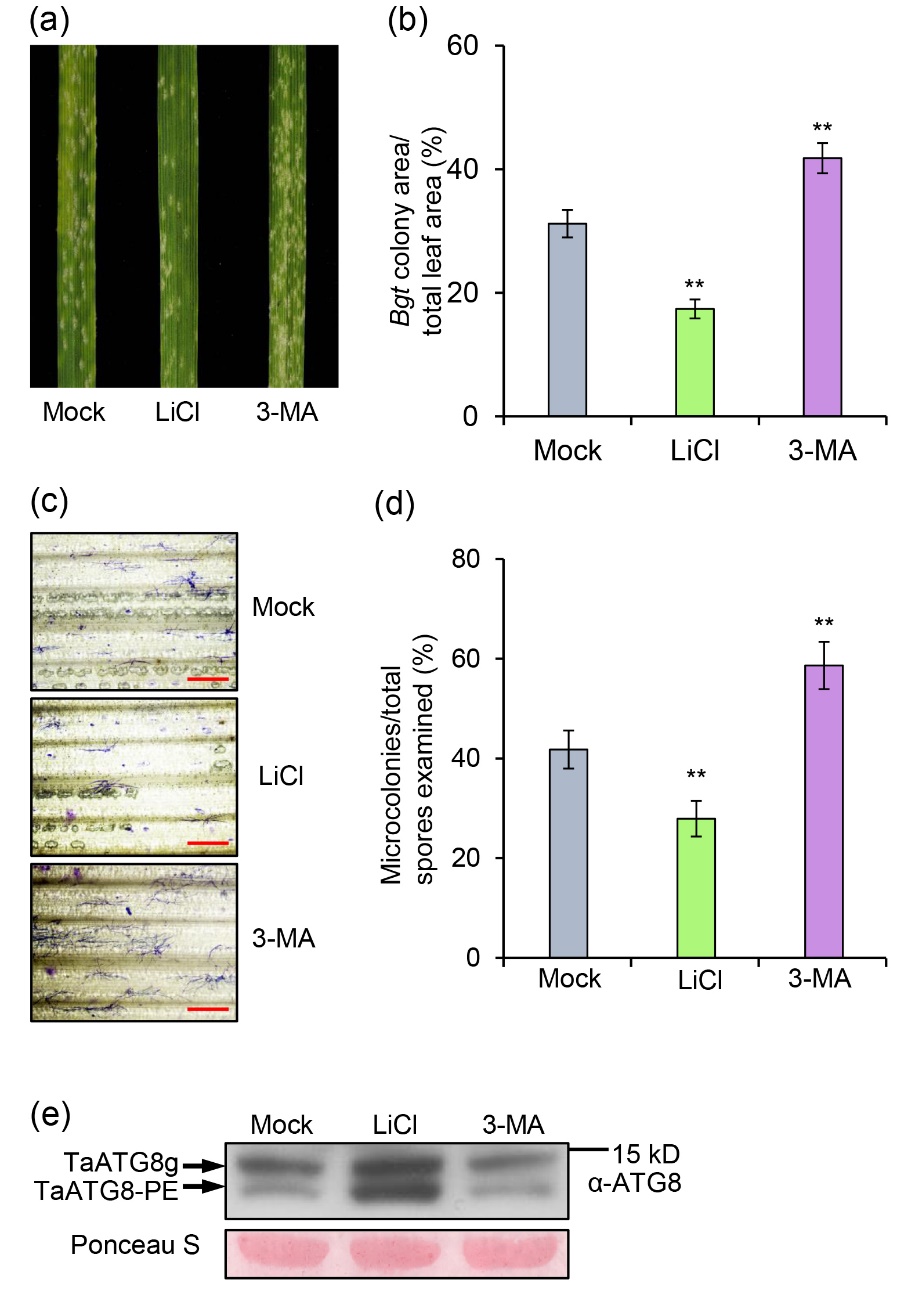


**Figure S6.** Effect of exogenous application of LiCl or 3-Methyladenine (3-MA) on wheat resistance to *Bgt*.

(a,b) *Bgt* colony growth on the leaves treated with LiCl (50 mM) or 3-MA (5 mM) at 8 dpi, as shown by photographs of *Bgt*-infected leaves (a) and quantitative comparison of the percentages of *Bgt* colony area (b). (c, d) Development of *Bgt* microcolonies on leaves treated with LiCl or 3-MA as shown by Coomassie blue staining (c) and quantitative comparison with the mock control (d). (e) TaATG8g and TaATG8-PE in leaves treated with LiCl or 3-MA revealed by immunoblotting.


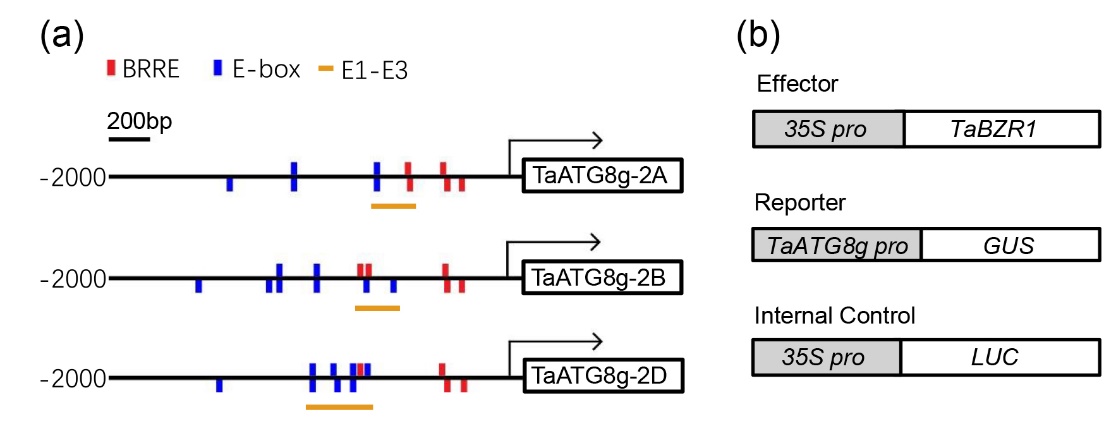


**Figure S7.** Structure of the *TaATG8* promoter and combinations of reporters and effector used in the transient expression assays.

(a) Distribution of cis-elements in *TaATG8* promoter. The E-box and BRRE elements were shown in blue and red blocks respectively. E1-E3, chromatin fragments used for ChIP–qPCR assays. (b) Diagrams showing the effector and reporter constructs used in transient expression assays.


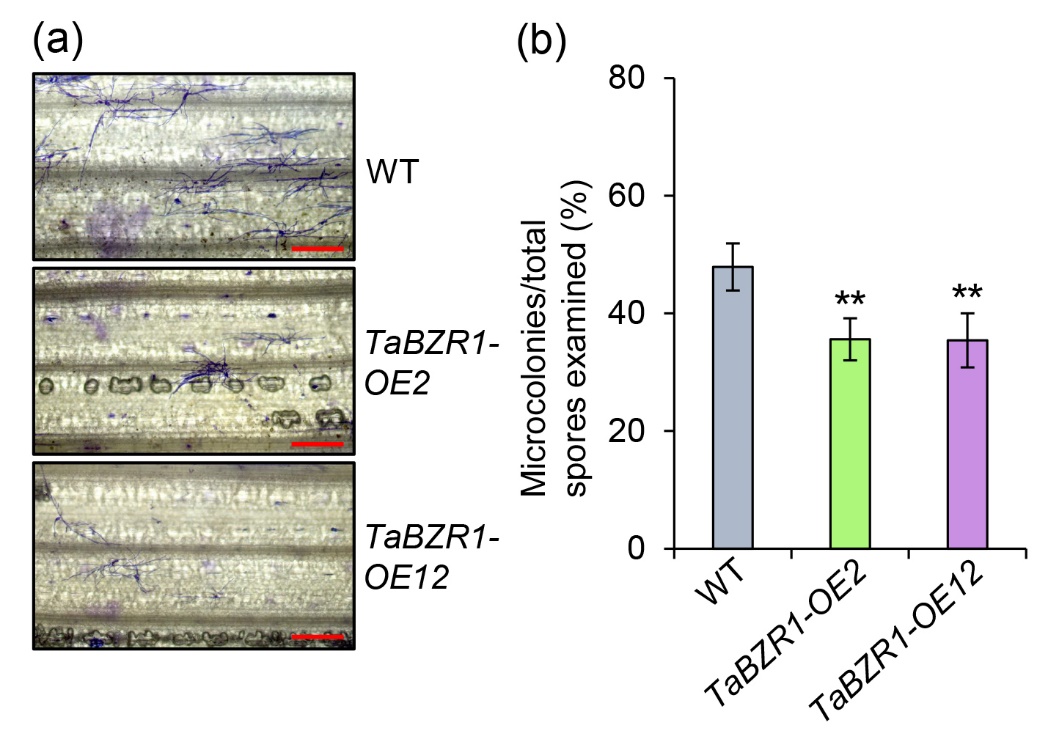


**Figure S8.** Effects of overexpressing *TaBZR1* on wheat defense against *Bgt*.

Reduced *Bgt* microcolonies development by overexpressing *TaBZR1*. Overexpression of *TaBZR1* depressed development of *Bgt* microcolonies (a, b). Data are means ± SD, n = 3. *P < 0.05; **P < 0.01 (Student's *t*‐test).


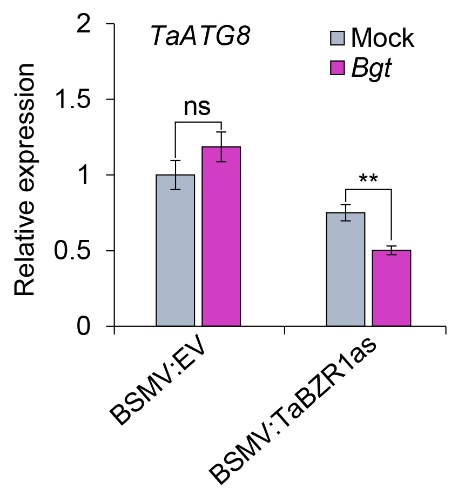


**Figure S9.** Transcript level of *TaATG8* in leaves of *TaBZR1* silenced plants.

Recombinant virus BSMV:TaBZR1as was used for silencing *TaBZR1*, with BSMV:EV as a control. *TaATG8* expression was detected at 36 hpi. Data are means ± SD, n = 3. **P < 0.01, according to Student's *t*‐test.


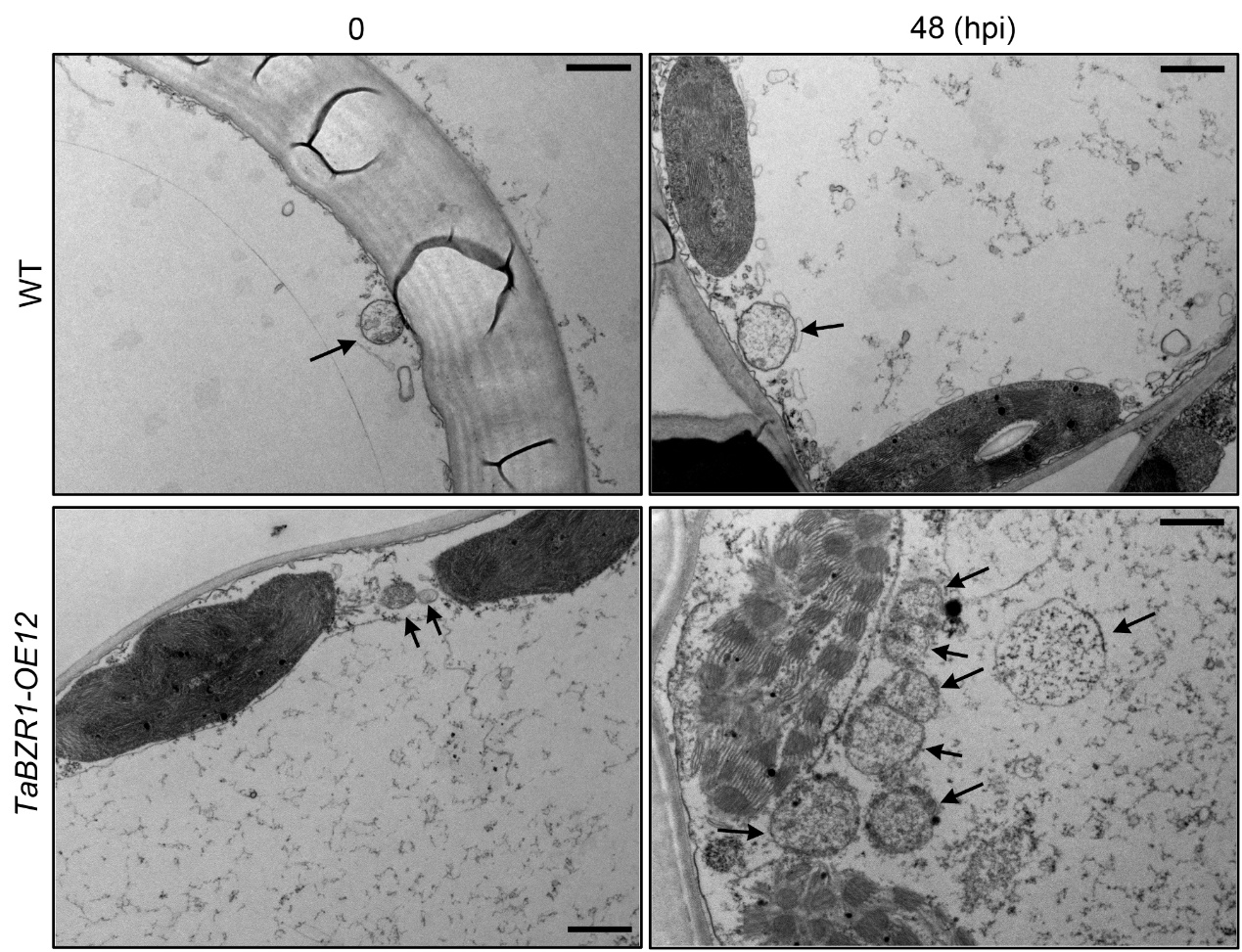


**Figure S10.** TEM observation of autophagic structures in *TaBZR1-OE12*.

TEM observation showed the increased autophagic bodies (arrows) in the *Bgt*-infected leaves of *TaBZR1-OE12*. Scale bar, 10 μm.


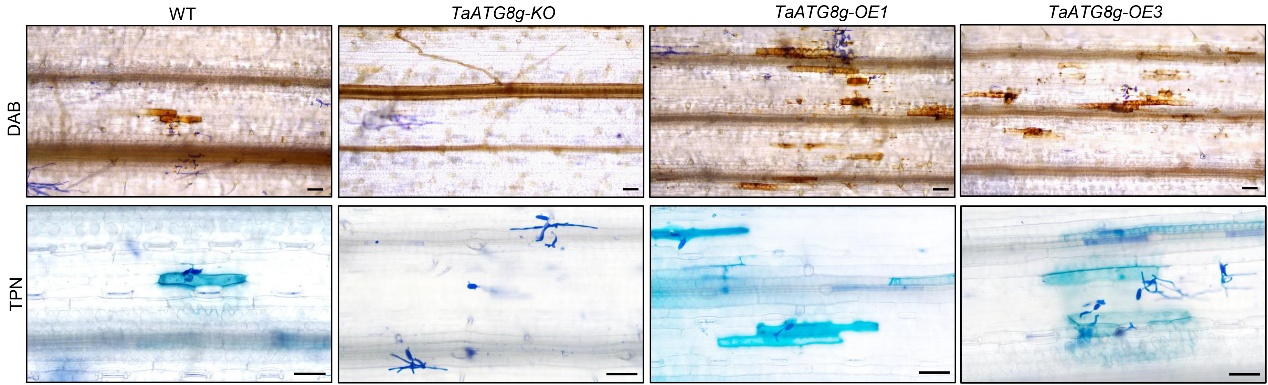


**Figure S11.** Detection of H_2_O_2_ accumulation and cell death in the *Bgt*-infected leaves of *TaATG8*-KO and *TaATG8*-OE lines.

DAB-Coomassie Blue (upper panel) and Trypan blue (lower panel) staining for detecting H_2_O_2_ accumulation and cell death, respectively, was performed on the *Bgt*-infected leaves. Scale bar, 200 μm.


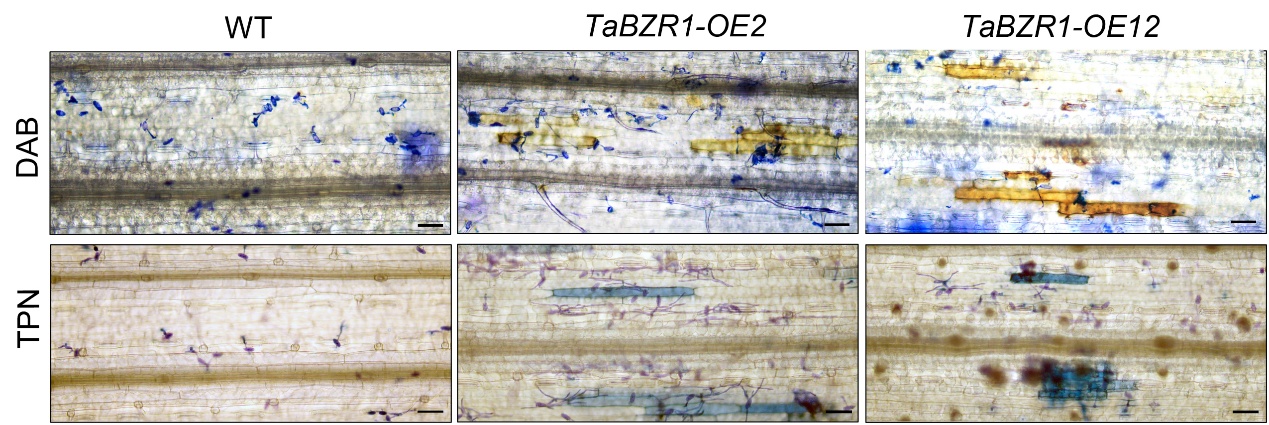


**Figure S12.** Detection of H_2_O_2_ accumulation and cell death in the *Bgt*-infected leaves of WT and *TaBZR1*-OE lines.

H_2_O_2_ accumulation and cell death revealed by DAB-Coomassie Blue (upper panel) and Trypan blue (lower panel) staining, respectively, was performed on the *Bgt*-infected leaves at 48 hpi. Scale bar, 200 μm.


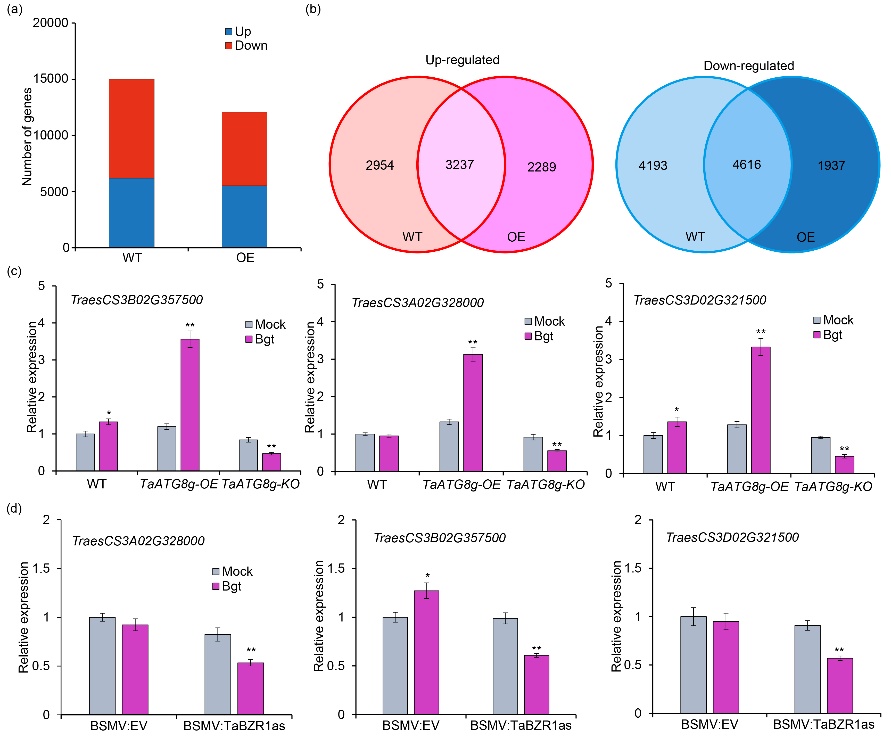


**Figure S13.** Analysis of the genes acted downstream of TaBZR1-*TaATG8g* module responding to *Bgt* inoculation.

(a) The numbers of up- and down-regulated genes triggered by *Bgt* infection in *TaATG8g-OE1* relative to WT. (b) The numbers of up- and down-regulated genes shared by wheat WT and *TaATG8g-OE1*. (c) Validation of expression of the three ERF genes in WT, *TaATG8g-OE1*, and *TaATG8g-KO* responding to *Bgt* infection. (d) Effects of silencing *TaBZR1* on the expression of the three ERF genes responding to *Bgt* infection. Data are means ± SD, n = 3. *P < 0.05; **P < 0.01, according to Student's *t*‐test.


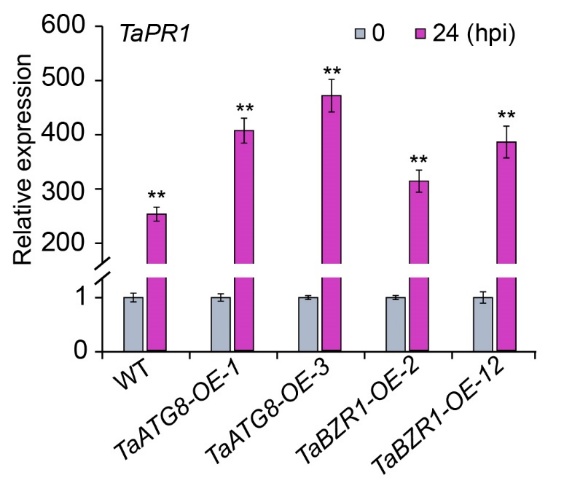


**Figure S14.** Transcript level of *TaPR1* in leaves of *TaATG8-* and *TaBZR1-* OE lines relative to WT.

*TaPR1* expression was detected at 24 hpi. Data are means ± SD, n = 3. **P < 0.01, according to Student's *t*‐test.


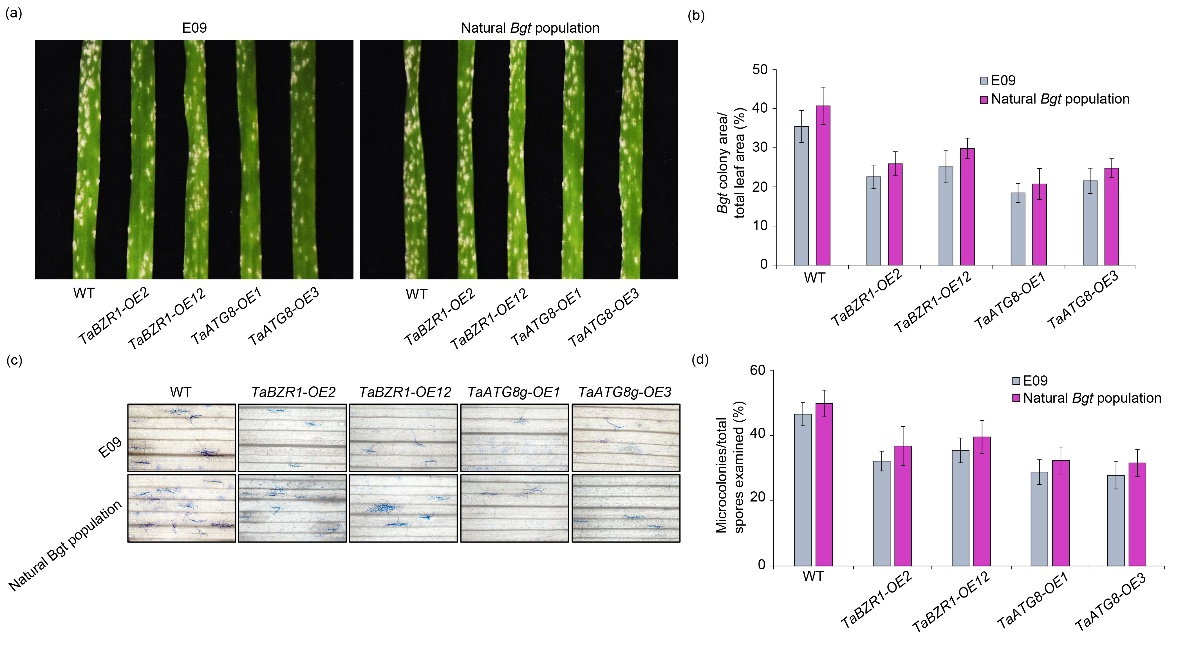


**Figure S15.** Infection phenotype of *TaATG8g-OE* and *TaBZR1-OE* lines to divergent *Bgt* isolates.

(a,b) *Bgt* colony growth on the leaves of WT, *TaATG8g-OE*, and *TaBZR1-OE* lines infected by E09 and a natural *Bgt* population at 8 dpi, as shown by photographs of *Bgt*-infected leaves (a) and quantitative comparison of the percentages of *Bgt* colony area (b). (c, d) Development of *Bgt* microcolonies on leaves of WT, *TaATG8g-OE*, and *TaBZR1-OE* lines infected by E09 and a natural *Bgt* population as shown by Coomassie blue staining (c) and quantitative comparison with the mock control (d).

| Primers used for gene cloning | | |
| --- | --- | --- |
| TaBZR1-F | | AAAAAGCAGGCTTTGACTTTATGACGTCGGGGGCGGCCC |
| TaBZR1-R | | AAGCTGGGTCTAGAGACTTTCTTTTCGAGCCGACGCCGAGCGTGAG |
| Primers used for qRT-PCR analysis | | |
| TaATG8g-2A-qRTF | | CCTCCCGTCGGTTGGAGAT |
| TaATG8g-2A-qRTR | | CCGGGACATCAGACTTATCAGCC |
| TaATG8g-2B-qRTF | | TTCCGTTGGAGATGGCCAAGAC |
| TaATG8g-2B-qRTR | | CCGGGACATCAGACTTATCAGCC |
| TaATG8g-2D-qRTF | | CCGTCGATTGTTTCCATCGATCGC |
| TaATG8g-2D-qRTR | | CCGGGACATCAGACTTATCAGCC |
| TaBZR1-2A-qRTF | | CCACACGACAGAGAAAGCTCAGC |
| TaBZR1-2A-qRTR | | CTCTCCTCTCTCTCCACTTGGCCTAG |
| TaBZR1-2B-qRTF | | GAAGAGAGGGGACGGAGAGAGAGAG |
| TaBZR1-2B-qRTR | | GCTTGTTGTTCTCCCGCTCCTTC |
| TaBZR1-2D-qRTF | | TGAGAGAGAGCAGCAGCGGAG |
| TaBZR1-2D-qRTR | | GCTTGTTGTTCTCCCGCTCCTTC |
| TaPR1-qRTF | | CTGGAGCACGAAGCTGCAG |
| TaPR1-qRTR | | CGAGTGCTGGAGCTTGCAGT |
| EMSA probes | | |
| TaATG8g-2A-Probe | | GGGTGGCGTGTCGCGGCTGTATTGCGTGCACAACTCGGGTGGCGTGTCGCGGCTGTATTGCGTGCACAACTC |
|  | |  |
| TaATG8g-2B-Probe | | GGCGACCGTGGGCACGTGACTGGGAGGGCGACCGTGGGCACGTGACTGGGAG |
|  | |  |
| TaATG8g-2D-Probe | | AGAATTCATATGAACATTGTCGTGTGCAGAGTTTAGTGCATGTGAATGTTAGAATTCATATGAACATTGTCGTGTGCAGAGTTTAGTGCATGTGAATGTT |
|  | |  |
| Primers used for ChIP-qPCR | | |
| ATG8-2A-ChIP-F | | CGAAACCGTGTGGCCTGTAG |
| ATG8-2A-ChIP-R | | TTTCTTTGCGTCCCATCTTGCG |
| 2Ack-ChIP-F | | GTTCTGGTGCGCTACTCCTCT |
| 2Ack-ChIP-R | | CCGACAACCACAAGCAATGGAATG |
| ATG8-2B-ChIP-F | | TTCGACAAAAAATGGCTGAGTTCATGC |
| ATG8-2B-ChIP-R | | CGAACCGGCGCTAGATCGTTC |
| 2Bck-ChIP-F | | CCAAGTCCTCTGAATCATGTTTGTTCC |
| 2Bck-ChIP-R | | CGGAGGCCCAACTCGAATTG |
| ATG8-2D-ChIP-F | CATCCATTAGATCACGTCATTTTGC | |
| ATG8-2D-ChIP-R | CACATGCACTAATGCATATTCCAGT | |
| 2Dck-ChIP-F | CCCCATCCCTTGAGGCTAGC | |
| 2Dck-ChIP-R | GAGACGGCCTGGTTTGAGAATG | |

**Table S1** Primers used in this study.

**SI-2 Supplementary materials and methods**

**Plant materials and powdery mildew isolates**

An F_2:6_ recombinant inbred lines (RILs) population derived from the cross of Citr 17345, carrying *Pm21,* and Yannong1212, highly susceptible to *Blumeria graminis* f.sp. *tritici* (*Bgt*) race E09, was used for gene expression analysis. The common wheat cultivar Fielder (susceptible to E09) was use as a recipient for developing the transgenic wheat lines overexpressing *TaATG8g*. The *TaBZR1* overexpressing lines were provided by Prof. Mingyi Bai from Shandong University.

***Bgt* inoculation and phenotyping**

*Bgt* infection and microscopy analyses were performed as previously reported (Zheng et al., 2019). Wheat seedlings at the one-leaf stage were inoculated with E09 spores. For analyzing the growth of *Bgt* colonies, the infected leaves were photographed at 8 d post-inoculation (dpi), with *Bgt* colony area determined using IMAGEJ software (<https://imagej.nih.gov/ij/>). For observing microcolonies development, the infected leaves were subjected to microcolonies staining using 0.6% (w/v) Coomassie brilliant blue at 72 h post-inoculation (hpi). In each inoculation, 10–12 leaves from at least 10 individual infected seedlings were examined, with the results used to calculate the percentage of microcolonies developing from the total number of examined *Bgt* spores. For detecting H_2_O_2_ accumulation, infected leaves were immediately soaked in a 1 mg ml^-1^ DAB solution (pH 3.8) for 16 h at 25 °C in dark, and then bleached in in boiling 95% ethanol. The bleached leaves subject to Coomassie blue staining before photographing. For observing cell death, infected leaves were incubated in a 0.4% Trypan blue dye for 10 min in boiling water, washed with sterile water, and then stained with Coomassie blue. The tested leaves were photographed using an Olympus BX53 microscope.

**qRT–PCR assays**

Total RNA isolation and first-strand cDNA synthesis were performed as described previously (Qi et al., 2021). Gene transcript levels were determined using the samples collected from at least 6 seedlings (including untreated controls and those treated for 24 h) by quantitative reverse transcription (qRT)-PCR with gene-specific primers (Supporting Information Table S1).

**Immunoblotting assay**

Total proteins were separated on SDS–PAGE gels, and protein blots were probed using an anti-TaATG8 antibody (Li et al., 2021).

**Virus-induced gene silencing**

Virus-induced gene silencing (VIGS) was performed as described previously (Zheng et al., 2019) using the barley stripe mosaic virus (BSMV) vector. Two vectors, pCaBS-γ:TaATG8as and pCaBS-γ:TaBZR1as harboring an antisense fragment derived from the coding sequence of *TaATG8* (120 bp) or *TaBZR1* (125 bp), were constructed. Together with pCaBS-α and pCaBS-β, the two recombinant viruses, BSMV:TaATG8as and BSMV:TaBZR1as, were formed using a RiboMAX Large Scale RNA Production-T7 kit with the addition of a cap analog (Promega, http://www.promega.com/), and used to silence endogenous *TaATG8* or *TaBZR1*.

**Overexpression of *TaATG8* in common wheat**

Transgenic overexpression of *TaATG8* in wheat was performed as described previously via *Agrobacterium tumefaciens*-mediated transformation (Zhang et al., 2018). Transgenic The construct pUbi:TaATG8-RFP was prepared with the vector pLGY-OE3 and the primers listed in Table S1. They were introduced into the immature embryos of Fielder by *Agrobacterium tumefaciens*-mediated transformation (Zhang et al., 2018). In the resulting transgenic plants, the transgene was detected via PCR and RT-qPCR using gene-specific primers (Supporting Information Table S1).

**Transient gene expression**

*Nicotiana benthamiana* protoplasts transient expression assays were performed as described previously (Zheng et al., 2021). The TaBZR1 effector construct and the GUS reporters driven by the TaATG8g promoter were combined and introduced into *Nicotiana benthamiana* protoplasts. A p35S:LUC vector was used as an internal control. The transfected protoplasts were cultured for 12 h in darkness. 4-Methylumbelliferone fluorescence and LUC luminescence were measured using a luminometer (Promega, USA). Relative GUS activity was determined by normalizing against the luciferase activity.

**Electrophoretic mobility shift assay (EMSA)**

EMSA was performed using a LightShift Chemiluminescent EMSA Kit (Thermo Fisher Scientific, USA) as described previously (Qi et al., 2021). Briefly, MBP and MBP-TaBZR1 were each expressed and purified from E. coli Rosetta (DE3) strain. Biotin-labeled synthetic oligonucleotides (Invitrogen, USA) were annealed with unlabeled oligonucleotides and then used as probes. The DNA probes were incubated with 100 ng of MBP or MBP-TaBZR1 protein in the binding buffer for 20 min. The resulting products were then subjected to native polyacrylamide gel electrophoresis, followed by transfer to a nylon membrane which was used for detection of EMSA signals according to the manufacturer’s instructions.

**Chromatin immunoprecipitation (ChIP)-quantitative (q) PCR analysis**

ChIP–qPCR assays were performed as described previously (Qi et al., 2021). WT Fielder and *TaBZR1-OE12* were used. Seedlings at the one-leaf stage were inoculated with or without E09 for 24 h. Nuclei and chromatin were isolated from each sample (3 g). The chromatin was sonicated and then immunoprecipitated with GFP-Trap beads or control empty beads at 4℃ overnight. The immunoprecipitated DNA was recovered and analyzed in triplicate by real-time qPCR with gene-specific primers (Supporting Information Table S1). Fold enrichment was calculated by comparison with the internal control performed by amplifying the Ubiquitin gene.

**Transmission Electron Microscopy (TEM) assay**

TEM observation was performed as described previously (Li et al., 2019). Control and *Bgt*-infected leaves at 48 hpi were cut into 1-2 mm segments and fixed in paraformaldehyde-glutaraldehyde fixative solution (2%/2.5%, pH 7.2). The fixed tissues were finally embedded in Epon 812. The embedding blocks were cut into 70-nm ultrathin sections examined using a Hitachi HT7700 electron microscope.

**Accession numbers**

Sequence data in this article can be found in the Ensembl Plants (https://plants.ensembl.org/index.html) under accession numbers: TaBZR1 (TraesCS2A02G187800, TraesCS2B02G219300, TraesCS2D02G199900), TaATG8g (TraesCS2A02G224000, TraesCS2B02G274200, TraesCS2D02G229900).

**References**

**Zhang, S., Zhang, R., Song, G., Gao, J., Li, W., Han, X., Chen, M., Li, Y., and Li, G.** (2018). Targeted mutagenesis using the Agrobacterium tumefaciens-mediated CRISPR-Cas9 system in common wheat. BMC Plant Biol. **18:** 302

**Zheng, H., Dong, L., Han, X., Jin, H., Wang, H., Yin, C., Han, Y., Li, B., Qin, H., Zhang, J., et al.** (2020) The TuMYB46L-TuACO3 module regulates ethylene biosynthesis in einkorn wheat defense to powdery mildew. New Phytologist, **225:** 2526-2541
